# A Mixture of Fermented Schizandrae Fructus Pomace and Hoveniae Semen cum Fructus Extracts Synergistically Protects against Oxidative Stress-Mediated Liver Injury

**DOI:** 10.3390/antiox12081556

**Published:** 2023-08-03

**Authors:** Jang-Soo Kim, Kyung-Hwan Jegal, Hye-Rim Park, Beom-Rak Choi, Jae-Kwang Kim, Sae-Kwang Ku

**Affiliations:** 1 Department of Anatomy and Histology, College of Korean Medicine, Daegu Haany University, Gyeongsan-si 38610, Republic of Korea; akamjnj@dhu.ac.kr (J.-S.K.); hrpark@nutracore.co.kr (H.-R.P.); 2 Department of Korean Medical Classics, College of Korean Medicine, Daegu Haany University, Gyeongsan-si 38610, Republic of Korea; jegalkh@dhu.ac.kr; 3 Nutracore Co., Ltd., Suwon-si 16514, Republic of Korea; brchoi@nutracore.co.kr; 4 Department of Physiology, College of Korean Medicine, Daegu Haany University, Gyeongsan-si 38610, Republic of Korea

**Keywords:** fermented Schizandrae Fructus pomace, Hoveniae Semen cum Fructus, carbon tetrachloride, acute liver injury, antioxidant, anti-inflammation

## Abstract

Schizandrae Fructus (SF) and Hoveniae Semen cum Fructus (HSCF) have long been used as medicinal herbs for treating various diseases in Asian traditional medicine. In the current study, we investigated the protective effect of fermented SF pomace and HSCF extract 1:1 (*w*:*w*) combination mixture (MSH) against carbon tetrachloride (CCl_4_)-induced acute liver injury mice. After MSH (50–200 mg/kg) oral administration for 7 consecutive days, animals were injected intraperitoneally with CCl_4_ (0.5 mL/kg). Histopathological observation revealed that administration of MSH synergistically decreased the degeneration of hepatocytes and the infiltration of inflammatory cells induced by CCl_4_. Moreover, MSH administration reduced the activities of alanine aminotransferase, aspartate aminotransferase, and γ-glutamyl transpeptidase in serum, and mitigated apoptotic cell death in hepatic parenchyma. In addition, MSH alleviated CCl_4_-mediated lipid peroxidation by restoring endogenous antioxidants capacities including glutathione contents, superoxide dismutase, and catalase activities. In vitro assessments using *tert*-butyl hydroperoxide-induced oxidative stress in HepG2 cells revealed that MSH protects hepatocytes by lowering ROS generation and lipid peroxidation via upregulating the transcriptional activity of nuclear factor erythroid-2-related factor 2 and the expression of antioxidant genes. Furthermore, MSH synergistically attenuated the expression of proinflammatory cytokines in CCl_4_-injured liver and lipopolysaccharide-stimulated RAW 264.7 cells. Taken together, these findings suggest that MSH has the potential to prevent acute liver damage by effectively suppressing oxidative stress and inflammation.

## 1. Introduction

In organisms relying on aerobic respiration, reactive oxygen species (ROS) is naturally generated as a byproduct of oxygen-utilizing energy metabolism. Normally, the ROS is responsible for physiological functions such as cellular signaling transduction, proliferation, and immune responses [1]. However, excessive production of free radical impairs the resilience of endogenous antioxidant system and triggers oxidative stress. Under such conditions, these free radicals provoke lipid peroxidation through oxidation of cellular polyunsaturated fatty acids. The formation of lipid peroxides exerts cytotoxic effect by altering the integrity of cellular membrane, and modifying the structure of biomolecules such as protein and nucleic acid [2]. Therefore, disruption of redox homeostasis is highlighted in the pathogenesis of various diseases like cardiovascular, neurodegenerative, and liver diseases [3,4].

The liver, as a primary organ responsible for detoxification, is regularly exposed to ROS generated during the biotransformation of xenobiotics including alcohol, drugs, and environmental pollutants [4]. When the accumulation of ROS exceeds the capacity of endogenous antioxidant system in liver, it can lead to death of hepatocytes. Additionally, inflammation plays an important role in propagating hepatic tissue injury. Research has provided evidence supporting the close correlation between oxidative stress and inflammation in the development of liver disease [5,6]. ROS can stimulate the production of inflammatory mediators by influencing intracellular signaling cascade within immune cells. Conversely, inflammatory cells contribute to an exacerbation of oxidative stress in the inflamed tissue [7]. Given the key role of sustained hepatic oxidative stress and inflammation in the onset and progression of liver disease, the development of therapeutic and preventive agents targeting these processes in the liver is considered crucial.

Various dietary plants and medicinal herbs that possess antioxidant and anti-inflammatory properties have been proposed as potential preventive and therapeutic agents for liver diseases. Schizandrae Fructus (SF), the fruit of *Schisandra chinensis* (Turcz.) Baill., called five flavor berry, has been used for treating gastrointestinal disorders, respiratory diseases, excessive sweating, and insomnia in East Asian traditional medicine [8]. Pharmacological studies revealed the beneficial effects of SF on alcohol and high fat diet-induced fatty liver, and drug-induced liver injuries [9,10,11]. Moreover, schizandrins and gomisin, biologically active components of *S. chinensis*, have been reported to possess protective effects against oxidative stress-mediated liver injury caused by carbon tetrachloride (CCl_4_) and ethanol through their antioxidant and anti-inflammatory effects [12,13,14,15,16]. In addition, Hoveniae Semen cum Fructus (HSCF), dried peduncle of *Hovenia dulcis* Thunb., has long been used as folk remedy for alcohol intoxication. Similar to its use in traditional medicine, preventive effects of HSCF against liver injury induced by hepatotoxins such as alcohol, CCl_4_, and D-galactosamine (D-gal)/lipopolysaccharide (LPS) have been researched [17,18]. Myricetin and dihydromyricetin, flavonoids identified in *H. dulcis* also exhibited antioxidant, anti-inflammatory, and anti-fibrotic activity in the liver of CCl_4_ intoxicated mice [19,20,21,22,23]. Although the hepatoprotective effects of both SF and HSCF have been suggested, a study attempting to elucidate the synergistic hepatoprotective effect of SF and HSCF combination therapy has not been conducted.

As a part of developing the hepatoprotective herbal agents, we discovered the mixtures of fermented SF pomace (fSFP) and HSCF (MSH) ameliorated CCl_4_-induced acute liver injury in the previous study [24]. Among the various combinations of ratios, 1:1 (*w*:*w*) MSH exhibited the most potent hepatoprotective effect against oxidative stress-mediated hepatic degeneration by enhancing endogenous antioxidant capacity including glutathione (GSH) content, superoxide dismutase (SOD), and catalase (CAT) activity. However, the previous study only confirmed the best ratio for liver protection effects, lacking verification of the concentration-dependent effect of MSH (1:1) and the molecular mechanism related to antioxidant and anti-inflammatory actions. Hence, this study examined the dose-dependent efficacy of MSH and explored its synergistic hepatoprotective effects in comparison to individual treatments of fSFP or HSCF against acute liver injury induced by CCl_4_. Additionally, we investigated the antioxidant and anti-inflammatory effects of MSH to unravel the underlying molecular mechanisms involved.

## 2. Materials and Methods

### 2.1. Materials and Reagents

Anti-4-hydroxynonenal (HNE) antibody was supplied from Abcam (Cambridge, UK). Anti-cleaved caspase-3 was obtained from Cell Signaling Technology (Beverly, MA, USA). Anti-cleaved poly(ADP-ribose) polymerase (PARP) antibody was purchased from Santa Cruz Biotechnology (Santa Cruz, CA, USA). Anti-nitrotyrosine (NT) was from Millipore (Temecula, CA, USA). Trolox was obtained from (Calbiochem, Darmstadt, Germany). 3-(4,5-Dimethylthiazol-2-yl)-2,5-diphenyltetrazolium bromide (MTT), CCl_4_, silymarin, *tert*-butyl hydroperoxide (*t*BHP), LPS (from O26:B6 *Escherichia coli*), 2′,7′-dichlorofluorescein diacetate (DCFH-DA), and other materials were purchased from Sigma-Aldrich (St. Louis, MO, USA).

### 2.2. Preparation of fSFP, HSCF, Silymarin, and MSH

fSFP, HSCF, and MSH (1:1) extract powders were prepared and supplied from Nutracore (Suwon, Korea). Briefly, raw materials (fSFP and HSCF each of 100 kg) were extracted with hot water, and then filtered. The resulting extracts were concentrated using a vacuum evaporator, and dried using a spray drier as indicated in manufacturing process (Figure S1). fSFP, HSCF, MSH and silymarin powders were dissolved in distilled water and preserved in 4 °C refrigerators until oral administration. For the cell treatment, MSH extract was dissolved in distilled water (at 50 mg/mL), and filtered using 0.2 µm syringe filter (Advantec, Tokyo, Japan). Schizandrin (a marker compound of fSFP) and myricetin (a marker compound of HSCF) contents in MSH were determined using high-performance liquid chromatographic (HPLC) analysis (Figure S2). Results showed MSH contained 0.6 mg/g of schizandrin and 0.17 mg/g of myricetin.

### 2.3. Animal Husbandry and Experiment

Male ICR mice were supplied from OrientBio (Seungnam, Korea), and maintained under 20–25 °C temperature, 30–35% relative humidity, and 12/12 h of light and dark cycle-controlled conditions. After acclimatization for 7 days, mice were allocated into the 8 groups (10 mice per group): vehicle, CCl_4_, CCl_4_ + silymarin, CCl_4_ + fSFP, CCl_4_ + HSCF, CCl_4_ + MSH200, CCl_4_ + MSH100, and CCl_4_ + MSH50. Mice were orally administered silymarin (200 mg/kg), fSFP (200 mg/kg), HSCF (200 mg/kg), MSH (50, 100, or 200 mg/kg), or vehicle once daily for a continuous period of 7 days. One hour after the last oral administration of test substances, CCl_4_ (0.5 mL/kg) dissolved in olive oil (19 of olive oil: 1 of CCl_4_, *v*:*v*) was injected intraperitoneally. The same volume of distilled water and olive oil was used for vehicle treatment to provide same restrain stresses of oral gavages. Twenty-four hours after CCl_4_ injection, the liver and blood were collected under isoflurane anesthesia (2–3%). All animal experiments of this report were conducted according to the international regulations of the usage and welfare of laboratory animal and were approved by the institutional Animal Care and Use Committee in Daegu Haany University (Approval No, DHU2021-023).

### 2.4. Measurement of Body and Liver Weight

Body weight of animals was measured once a day using an electronic balance (XB320M, Precisa, Dietikon, Switzerland). Liver weight was measured at sacrifice of animals, and the relative liver weight represents the ratio of liver weight to total body weight of each animal.

### 2.5. Histopathological Analysis

Left lateral lobes of livers were fixed in 10% neutral buffered formalin. Cross trimmed hepatic tissues of each liver were embedded in paraffin, and serially sectioned (3–4 µm thick) using a microtome (RM2255, Leica Biosystems, Nussloch, Germany). For general histopathological observation, hematoxylin and eosin (Sigma-Aldrich) stained sectioned were assessed under a light microscope (Nikon, Tokyo, Japan) at ×100 or ×400 magnification. The number of hepatocytes with degenerative changes including necrosis, cellular swelling, and severe fatty changes, and of infiltrated inflammatory cells in hepatic parenchyma were counted using an automated image analyzer (*i*Solution FL ver 9.1; IMT *i*-solution Inc., Burnaby, BC, Canada). Hepatic damage was evaluated by the modified Histological Activity Index (HAI) grading scores that included assessment of confluent necrosis, focal lytic necrosis, apoptosis, and focal and portal inflammation, according to established histopathological scoring system [25]. All histopathological assessment were conducted by a certified pathologist who was blinded to the group distribution.

### 2.6. Immunohistochemistry

Hepatic tissue sections were heated (95–100 °C) in citrate buffers (pH 6.0, 10 mM) for antigen retrieval and incubated in methanol and 0.3% H_2_O_2_ (30 min) for blocking endogenous peroxidase activity. After incubation with anti-cleaved caspase-3 (1:400, Cell Signaling Technology, #9661), anti-cleaved PARP (1:100, Santa Cruz Biotechnology, #sc-23461), anti-4-HNE (1:100, Abcam, #ab46545), and anti-NT (1:200, Millipore, #06-284) antibodies, respectively, at 4 °C for overnight in humidified chamber, primary antibodies immunoreactive cells were observed using avidin-biotin-peroxidase complex (ABC) based immunohistochemistry method. Stained sections were observed under a microscope (Nikon) at ×200 magnification, and immuno-positive cells around central vein were counted using an automated image analyzer (IMT *i*-solution Inc.).

### 2.7. Liver Function Tests

The blood obtained from the vena cava underwent centrifugation to separate the serum. The activity of alanine aminotransferase (ALT) and aspartate aminotransferase (AST), and γ-glutamyl transpeptidase (GGT) in serum was assessed using biochemistrical autoanalyzer (Dri-Chem NX500i, Fuji Medical System, Tokyo, Japan) with Fuji Dri-Chem GPT/ALT-PIII (#3250), GOT/AST-PIII (#3150), and GGT-PIII (#3050) slides (Fuji Medical System).

### 2.8. Quantification of Lipid Peroxidation

To determine the extent of lipid peroxidation in the liver, hepatic malondialdehyde (MDA) contents were measured by using thiobarbituric acid (TBA) test. Briefly, after homogenization in ice-cold 0.01 M Tris-HCl (pH 7.4), hepatic tissues were centrifuged at 12,000× *g* for 15 min. After reaction with TBA, forming MDA-TBA adduct was estimated by measuring absorbance at 525 nm. MDA contents were normalized with total protein contents of liver homogenates and represented as nM MDA/mg protein.

To measure the lipid peroxidation in HepG2 cells, we conducted a flow cytometry using C11-BODIPY (Thermo Fisher Scientific, Rockford, IL, USA) dye. Cells were pretreated with MSH (100–1000 µg/mL, 1 h), and subsequently incubated with *t*BHP (200 µM) and C11-BODIPY for 3 h. After treatment, fluorescence intensity was measured at 485/530 nm (excitation/emission wavelengths) using an automated microplate reader (Infinite 200 PRO, Tecan, Männedorf, Switzerland). Additionally, the treated cells were detached by trypsinization, and the percentage of cells expressing high intensity of C11-BODIPY was analyzed using a flow cytometry (Accuri™C6 Plus; BD Biosciences, San Diego, CA, USA). A total of 10,000 events were recorded in each experiment.

### 2.9. Measurement of Endogenous Antioxidant System Capacities in Hepatic Tissue

GSH contents, SOD and CAT activities in hepatic tissue homogenates were measured as following established methods in previous research [6]. Each measured value was normalized with total protein contents of tissue homogenates.

### 2.10. Measurement of Radical Scavenging Activity

DPPH (2,2-diphenyl-1-picrylhydrazyl) assay was conducted to measure radical scavenging activity. Briefly, dissolved test substances in 50 µL of distilled water were reacted with 50 µL of DPPH solution (150 µM, in ethanol) for 30 min in the dark. Absorbance at 517 nm was monitored using an automated microplate reader (Synergy HTX Multimode Reader, BioTek, Winooski, VT, USA), and each measured value was subtracted with the value of blank well (without test samples). Radical scavenging activity (%) was calculated as follows:

Radical scavenging activity (%) = [1 − (Absorbance of test group/Absorbance of control group)] × 100

### 2.11. Cell Culture and Treatments

HepG2 (a human hepatocarcinoma cell line) and RAW 264.7 cells (a murine macrophage cell line) were supplied from American Type Culture Collection (ATCC; Rockville, MD, USA). Cells were maintained in Dulbecco’s modified Eagle’s medium (HyClone Laboratories, Logan, UT, USA) containing 10% fetal bovine serum (FBS) and 1% Antibiotic-Antimycotic (Thermo Fisher Scientific), under humidified conditions with 5% CO_2_, at 37 °C temperature. To assess the effect of MSH on *t*BHP-induced oxidative stress, HepG2 cells grown in an appropriate multi-well plate were serum-deprived for 12 h, and pretreated with MSH (30–1000 mg/mL) for 1 h, and then exposed to *t*BHP (200 µM) for 3–12 h. To measure the expression of inflammatory mediators, RAW 264.7 cells grown in 96 well plate at a density of 1 × 10^4^ per well were pretreated with MSH (100–1000 mg/mL) in serum-depleted medium for 1 h, and then exposed to LPS (1 µg/mL) for 18 h. Details of cell treatment was presented in the description of each experiment.

### 2.12. Cell Viability Assay

To determine cytotoxic or cytoprotective effects of MSH, cell viability was measured using MTT assay. HepG2 cells were plated at a density of 1 × 10^4^ per well in 96 well plate, and serum-starved for 12 h before the treatment. To assess the cytotoxic effect of MSH, cells were treated with MSH (30–1000 mg/mL) for 24 h. To examine cytoprotective effect of MSH, cells were pretreated with MSH (30–1000 mg/mL) for 1 h, and then exposed to *t*BHP (200 µM) for 12 h. After treatment, cells were further incubated with MTT solution (final conc.: 0.1 µg/mL) for 4 h. Formed purple-colored formazan products were dissolved by 100 µL of dimethyl sulfoxide. Absorbance at 570 nm was detected using an automated microplate reader (BioTek). Relative cell viability (%) was calculated as follows:

Relative cell viability (%) = [(Absorbance of test group)/(Absorbance of control group)] × 100

### 2.13. Measurement of ROS Production

HepG2 cells were cultured in a 96 well (1 × 10^4^ per well), black-wall, transparent-bottom plate. After serum deprivation, cells were pretreated with 100–1000 µg/mL of MSH for 1 h. Then, cells were further exposed to *t*BHP (200 µM) for 3 h, in the presence of DCFH-DA (10 µM). Florescence intensity at emission 485 nm and excitation 530 nm was detected using an automated microplate reader (Tecan).

### 2.14. Reporter Gene Assay

Recombinant HepG2 cells, expressing antioxidant response element (ARE)-driven luciferase stably, were plated at 12 well plate (5 × 10^5^ cells/well). After serum deprivation, cells were treated with MSH (100–1000 mg/mL) for 18 h, and then luciferase assay reagent (Promega, Madison, WI, USA) was added to cell lysates. Luciferase activity was measured by detecting luminescence using a GloMax^®^ 20/20 luminometer (Promega). Each measured luminescence intensity was normalized with a protein content of each sample.

### 2.15. Quantitative Polymerase Chain Reaction (qPCR)

Total RNA was extracted from hepatic tissues by TRIzol reagent (Thermo Fisher Scientific). cDNA was synthesized using the High-Capacity cDNA Reverse transcription kit (Thermo Fisher Scientific). Real-time PCR was performed using CFX96™ real-time system (Bio-Rad, Hercules, CA, USA). The expression of *actin beta* or *glyceraldehyde 3-phosphate dehydrogenase* mRNA was used for normalization of each mRNA expression, and all relative mRNA expressions were represented utilizing the 2^−ΔΔCt^ method [26].

### 2.16. Measurement of Inflammatory Mediators

To measure expressions of inflammatory mediators in hepatic tissues, liver (right lobe) was homogenized in ice-cold RIPA (radioimmunoprecipitation assay) buffer using a bead homogenizer (taco™Prep Bead Beater, GeneReach Biotechnology Corp., Taichung, Taiwan). After centrifugation at 20,000× *g* for 20 min, the expression of tumor necrosis factor-α (TNF-α), interleukin (IL)-1β, and IL-6 in the resulting supernatant were measured by commercial enzyme-linked immunosorbent assay (ELSIA) kit (Mybiosource, San Diego, CA, USA), following manufacturer’s instructions.

In the case of in vitro experiment, nitric oxide (NO), prostaglandin E_2_ (PGE_2_), TNF-α, IL-1β, IL-6, and monocyte chemoattractant protein-1 (MCP-1) expressions in conditioned media were quantified. To measure NO, conditioned media (100 µL) was reacted with the same volume of Griess reagent, and absorbance at 540 nm was detected using an automated microplate reader (BioTek). Expressions of proinflammatory cytokines including TNF-α, IL-1β, IL-6, MCP-1 (BD Biosciences), and PGE_2_ (R&D Systems, Minneapolis, MN, USA) in conditioned media were measured using commercial ELISA kit.

### 2.17. Statistical Analysis

All numerical values were represented as mean ± standard deviation of 10 mice. According to variance homogeneity, one-way ANOVA (as a parametric method) or Kruskal–Wallis H test (as a non-parametric method) was conducted to determine significant differences among the experimental groups. If a significance was observed, Tukey’s HSD or Mann–Whitney U test was followed to determine the specific group comparison. *p* values under 0.05 was considered statistically significant. All statistical analyses were performed using SPSS 14.0 (IBM, Armonk, NY, USA).

## 3. Results

### 3.1. MSH Synergistically Protects the Liver against CCl_4_

**Figure 1:**
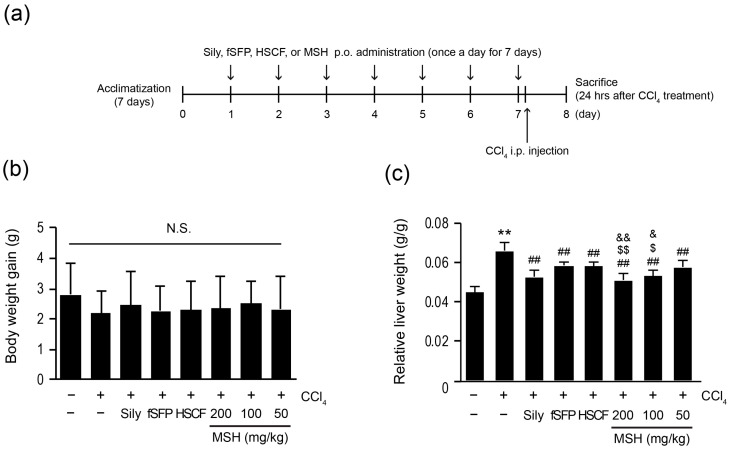
Effect of combination mixture of fermented Schizandrae Fructus pomace (fSFP) and Hoveniae Semen cum Fructus (HSCF) on body weight gain and relative liver weight in CCl_4_-treated mice. (**a**) Scheme of animal experiment. Mice (10/group) were administered with silymarin (Sily, 200 mg/kg), fSFP (200 mg/kg), HSCF (200 mg/kg), or three doses of MSH (mixture of fSFP and HSCF; 200, 100, 50 mg/kg) for 7 consecutive days. One hour after the last administration of test materials, mice were intraperitoneally injected with CCl_4_ (0.5 mL/kg). (**b**) Body weight gain was calculated by subtracting the mice body weight on day 0 from those of day 7. (**c**) Relative liver weight. Absolute liver weight was divided by body weight on day 7. All values were represented as mean ± standard deviation (SD) of ten mice. Significant versus vehicle group, ** *p* < 0.01; versus CCl_4_-injected group, ^##^ *p* < 0.01; versus fSFP-treated group, ^$^ *p* < 0.05, ^$$^ *p* < 0.01; versus HSCF-treated group, ^&^ *p* < 0.05, ^&&^ *p* < 0.01. N.S., not significant.

To investigate the hepatoprotective effects of MSH, mice were administered with fSFP (200 mg/kg), HSCF (200 mg/kg), or MSH (50, 100, and 200 mg/kg) for 7 days, once daily. Silymarin (200 mg/kg) was also administered for 7 days as a positive drug because it showed the consistent hepatoprotective effect in various hepatoxin-induced liver injury animal models, including CCl_4_ and ethanol at a concentration of 200 mg/kg [24,27,28]. One hour after the last administration of test materials, CCl_4_ (0.5 mL/kg) was injected to provoke liver injury (Figure 1a). Twenty-four hours after CCl_4_ injection, all mice were sacrificed for further analysis. Statistically significant differences were not observed in body weight change (between the beginning and the end of experiment) among all experimental groups (Figure 1b). As previously reported [6], CCl_4_ injection, compared to vehicle-treated group, significantly increased the relative liver weight (liver weight/body weight, g/g). However, CCl_4_-mediated elevation of the relative liver weight was significantly ameliorated by MSH (50–200 mg/kg), fSFP, HSCF, and silymarin administration. Furthermore, the extent of relative liver weight reduction achieved through MSH (100, 200 mg/kg) administration was significantly greater than reductions observed with either fSFP or HSCF single administration (Figure 1c).

**Figure 2:**
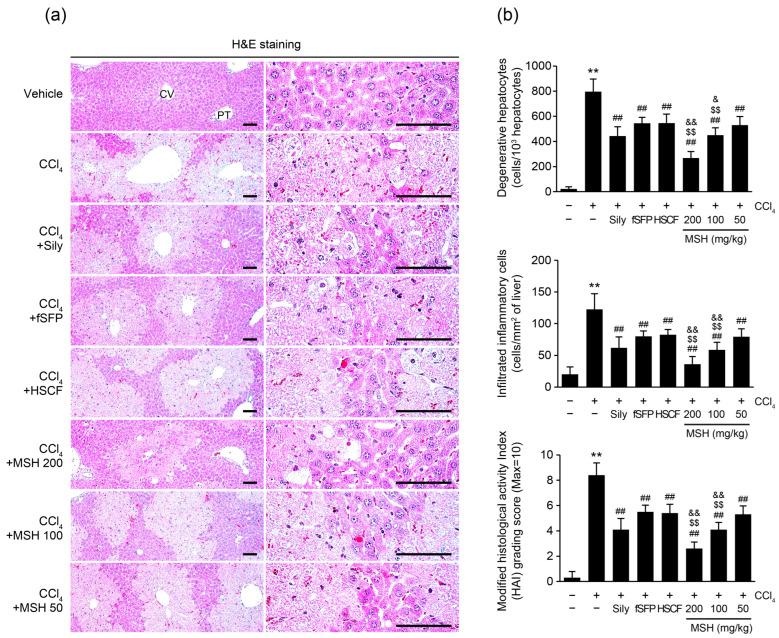
MSH protects the liver from CCl_4_-induced hepatic damage. (**a**) Representative hepatic tissue profiles. Hepatic tissues were stained with hematoxylin and eosin. Scale bar indicates 200 µm. (**b**) Histopathological analysis. The number of degenerative hepatocytes (upper), the number of infiltrated inflammatory cells (middle), and modified HAI grading scores (lower). Significant versus vehicle group, ** *p* < 0.01; versus CCl_4_-injected group, ^##^ *p* < 0.01; versus fSFP-treated group, ^$$^ *p* < 0.01; versus HSCF-treated group, ^&^ *p* < 0.05, ^&&^ *p* < 0.01. CV, central vein; PT, portal triad.

Histopathological observation using hematoxylin and eosin-staining indicated that CCl_4_ significantly increased the number of degenerated hepatocytes and infiltrated inflammatory cells in hepatic parenchyma. In addition, modified HAI scores, representing confluent necrosis, focal lytic necrosis, apoptosis, and portal inflammation in hepatic tissue, were also increased by CCl_4_ challenge. However, these histopathological changes of the liver by CCl_4_ injection were significantly restored by administration of MSH (50–200 mg/kg), fSFP, HSCF, and silymarin. Especially, the degree of reductions in the numbers of degenerative hepatocytes, infiltrated inflammatory cells, and the HAI score against CCl_4_-induced hepatic damages by 100, 200 mg/kg of MSH administration were significantly greater than those by either fSFP or HSCF single administration group (Figure 2).

### 3.2. MSH Synergistically Ameliorates Hepatocyte Apoptosis in CCl_4_-Induced Liver Injury

**Figure 3:**
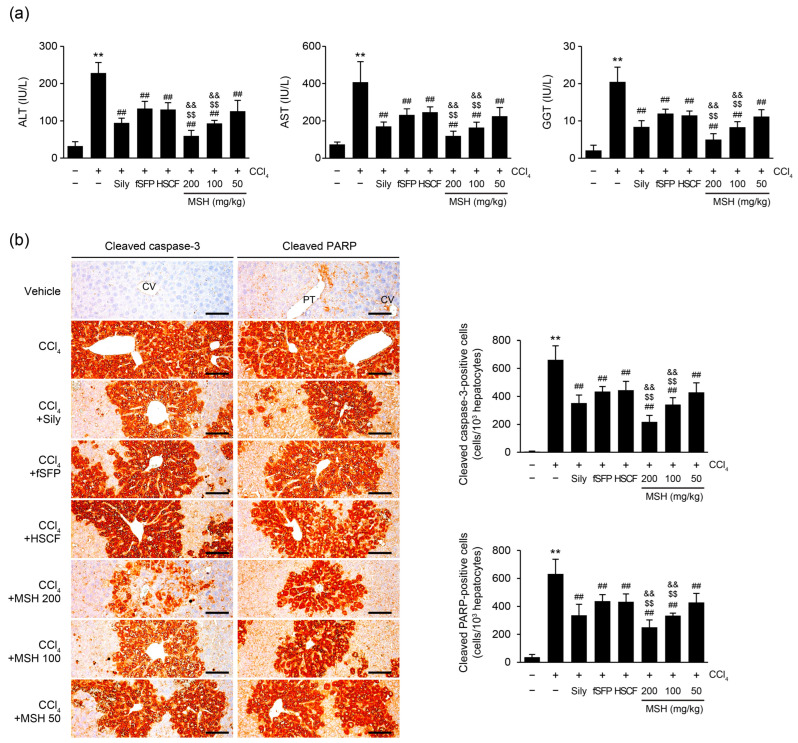
MSH ameliorates CCl_4_-induced apoptosis of hepatocytes. (**a**) Serum chemistry. Alanine aminotransferase (ALT), aspartate aminotransferase (AST), and γ-glutamyl transpeptidase (GGT) activities in serum. (**b**) Representative profiles of immunohistochemistry. Hepatic tissues were stained with anti-cleaved caspase-3 and anti-cleaved PARP antibodies. Scale bars indicate 200 µm. Significant versus vehicle group, ** *p* < 0.01; versus CCl_4_-injected group, ^##^ *p* < 0.01; versus fSFP-treated group, ^$$^ *p* < 0.01; versus HSCF-treated group, ^&&^ *p* < 0.01. CV, central vein; PT, portal vein.

To investigate the protective effect of MSH on CCl_4_-induced hepatocytes death, activities of serum markers including ALT, AST, and GGT that indicate damage to hepatocytes were measured (Figure 3a). As a result, administration of silymarin, fSFP, HSCF, and all doses of MSH (50–200 mg/kg) significantly lowered the increased activities of ALT, AST, and GGT in the serum by CCl_4_. Especially, 100 and 200 mg/kg of MSH administration exerted the more significant inhibitory effects on the elevation of ALT, AST, and GGT activities than fSFP or HSCF single administration. Next, we evaluated the prevalence of apoptosis in hepatic parenchyma by immunohistochemistry staining using anti-cleaved caspase-3 and anti-cleaved PARP antibodies. The numbers of cleaved caspase-3 and cleaved PARP-positive cells, which reflect apoptotic cell death, were significantly increased by CCl_4_ injection in hepatic tissue. However, administration of silymarin, fSFP, HSCF, and MSH (50–200 mg/kg) induced significant reductions in the numbers of cleaved caspase-3 and cleaved PARP-positive cells, compared to CCl_4_ injected group. Moreover, the reductions in the numbers of cleaved caspase-3 and cleaved PARP-positive cells achieved by MSH (100 and 200 mg/kg) administration was significantly greater than those observed in either fSFP or HSCF single administration (Figure 3b).

### 3.3. MSH Protects the Liver against CCl_4_-Induced Oxidative Stress by Enhancing Endogenous Antioxidant Capacity

**Figure 4:**
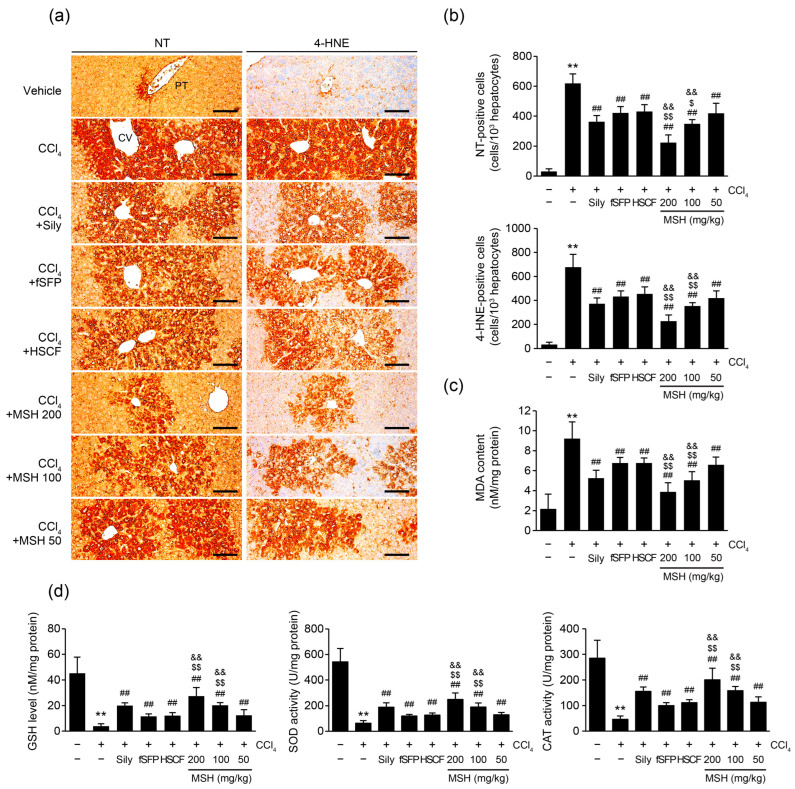
MSH attenuates CCl_4_-induced oxidative stress by enhancing antioxidant capacity in the liver. (**a**) Representative profiles of immunohistochemistry using anti-nitrotyrosine (NT), and anti-4-hydroxynonenal (4-HNE) antibodies. Scale bar indicates 200 µm. (**b**) The numbers of NT- and 4-HNE-positive cells. (**c**) Hepatic malondialdehyde (MDA) contents. MDA contents in liver homogenates were measured. (**d**) Endogenous antioxidant capacity. Glutathione (GSH) level, superoxide dismutase (SOD) activity, and catalase (CAT) activity in hepatic tissue were measured. Significant versus vehicle group, ** *p* < 0.01; versus CCl_4_-injected group, ^##^ *p* < 0.01; versus fSFP-treated group, ^$^ *p* < 0.05, ^$$^ *p* < 0.01; versus HSCF-treated group, ^&&^ *p* < 0.01. CV, central vein; PT, portal vein.

To explore whether the protective effect of MSH against CCl_4_-induced hepatocytes damage was mediated by mitigating oxidative stress in hepatic tissue, we measured the products of cellular oxidative stress by immunohistochemistry staining with anti-NT (a marker of nitrosative stress), 4-HNE (a marker of lipid peroxidation) antibodies. As is known, CCl_4_ injection increased the numbers of NT- and 4-HNE-positive cells in the liver, indicating that CCl_4_ caused oxidative stress in hepatic tissue. By contrast, silymarin, fSFP, HSCF, and MSH (50–200 mg/kg) significantly decreased the number of NT- and 4-HNE-positive cells. Moreover, MSH administration (100, 200 mg/kg) showed a significant additional inhibitory effect on CCl_4_-induced oxidative stress in hepatic tissue, compared to fSFP and HSCF single administration (Figure 4a,b). In addition, liver MDA content, another biomarker of lipid peroxidation, was assessed using hepatic tissue homogenates. Elevation of MDA content in liver by CCl_4_ injection was significantly reduced by MSH (50–200 mg/kg) administration, and the extent of MDA reduction in 100 and 200 mg/kg of MSH administration was significantly greater than that observed in fSFP or HSCF single administration (Figure 4c). Next, we investigated the effect of MSH on GSH level (an endogenous antioxidant) and SOD and CAT activities (endogenous antioxidant enzymes). CCl_4_ injection significantly depleted GSH level, and decreased SOD and CAT activities in liver homogenates. However, administration of silymarin, fSFP, HSCF, and three doses of MSH (50–200 mg/kg) significantly restored GSH level, and enzyme activities of SOD and CAT. Among the three doses of MSH administration, 100 and 200 mg/kg of MSH treatment showed significantly greater restorative effect on GSH level, SOD and CAT activities than either fSFP or HSCF single administration (Figure 4d).

### 3.4. MSH Protects Hepatocytes against tBHP-Induced Oxidative Stress via Activation of Nuclear Factor Erythroid 2-Related Factor 2 (Nrf2) Signaling Pathway

**Figure 5:**
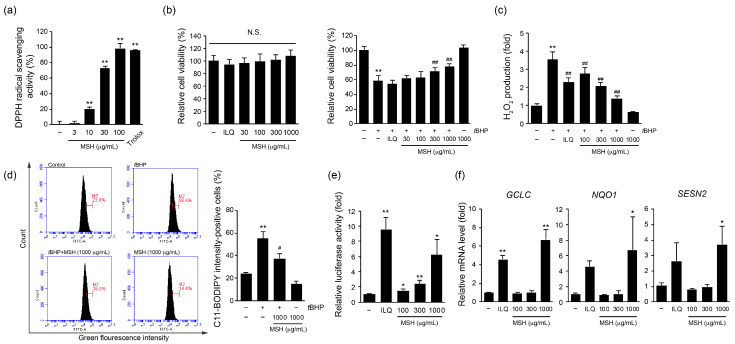
Hepatoprotective effect of MSH against *tert*-butyl hydroperoxide (*t*BHP)-induced oxidative stress in HepG2 cells. (**a**) DPPH radical scavenging activity. (**b**) Cell viability. Relative cell viability was measured by MTT assay. HepG2 cells were treated with 30–1000 µg/mL of MSH, or 10 µM of isoliquiritigenin (ILQ) for 24 h (left). HepG2 cells were pretreated with 30–1000 µg/mL of MSH for 1 h, and subsequently exposed to 200 µM of *t*BHP for 12 h (right). (**c**) ROS production. MSH (30–1000 µg/mL, 1 h) pretreated HepG2 cells were exposed to *t*BHP for 3 h, in the presence of DCFH-DA (10 µM). (**d**) Lipid peroxidation. HepG2 cells were treated as indicated in (**c**), with presence of C11-BODIPY. The percentage of cells with high green fluorescence intensity was analyzed by a flow cytometry. (**e**) Antioxidant response element (ARE)-driven luciferase activity. Recombinant HepG2 cells, which stably transfected with ARE-driven reporter gene, were incubated with 100–1000 µg/mL of MSH, or ILQ (10 µM) for 18 h, and then relative luciferase activity in cell lysates were measured. (**f**) Antioxidant genes expression. HepG2 cells were incubated with MSH (30–1000 µg/mL) or ILQ (10 µM) for 12 h. mRNA levels of *GCLC*, *NQO1*, and *SESN2* genes were measured by real-time qPCR. Relative mRNA expression of each gene was normalized by expression of *GAPDH*. All values were represented as mean ± SD of more than three separate experiments. Significant versus vehicle-control group, * *p* < 0.05, ** *p* < 0.01; versus *t*BHP-treated group, ^#^ *p* < 0.05, ^##^ *p* < 0.01. N.S., not significant.

Next, we investigated the antioxidant effect of MSH using in vitro experiments to elucidate molecular mechanisms involved. First, radical scavenging effect of MSH was evaluated using DPPH. MSH showed significant and the dose-dependent DPPH scavenging effect at 10–100 µg/mL (Figure 5a). At the dose of 100 µg/mL, radical scavenging activity of MSH was comparable to that of 100 µM trolox. Moreover, MSH showed no cytotoxic effect at range of 30–1000 µg/mL in HepG2 cells (Figure 5b, left). To evaluate hepatoprotective effect of MSH against oxidative stress, we used *t*BHP as oxidative stress inducer, which widely used for screening of hepatoprotective herb and phytomedicine [29]. After pretreatment with MSH (30–1000 µg/mL, 1 h), HepG2 cells were further exposed to *t*BHP (200 µM, 12 h). Compared to vehicle-treated control, *t*BHP treatment significantly decreased the cell viability. However, pretreatment with MSH (30–1000 µg/mL) increased the viability of HepG2 cells in a dose-dependent manner. Statistical significance was observed in 300 and 1000 µg/mL of MSH treatment, compared to *t*BHP alone treated cells (Figure 5b, right). To investigate if the cytoprotective effect of MSH against oxidative stress induced by *t*BHP is attributed to the reduction in intracellular ROS generation, we examined H_2_O_2_ production in *t*BHP-stimulated HepG2 cell using DCFH-DA. As a result, MSH pretreatment (300, 1000 µg/mL) significantly decreased *t*BHP (200 µM, 3 h)-induced H_2_O_2_ production (Figure 5c). Furthermore, we evaluated the effect of MSH on lipid peroxidation using C11-BIODIPY. The percentage of cells exhibiting high green fluorescence intensity, an indicator of lipid peroxidation intensity within cells, was reduced by MSH (1000 µg/mL) pretreatment (Figure 5d). To find the molecular mechanisms involved in antioxidative stress effect of MSH, luciferase activity using ARE-driven reporter gene was measured. ARE refers to the domain located in the promoter region of antioxidant genes, which are well-known binding sites for Nrf2 as a transcriptional factor. Compared to vehicle-treated cells, MSH (100–1000 µg/mL) treatment significantly increased ARE-driven luciferase activities in a dose-dependent manner (Figure 5e). Accordingly, 1000 µg/mL of MSH treatment (12 h) significantly increased mRNA expression of antioxidant genes including *glutamate-cysteine ligase catalytic subunit* (*GCLC*), *NAD(P)H quinone oxidoreductase 1* (*NQO1*), and *sestrin2* (*SESN2*), all of which are known as target genes of Nrf2 (Figure 5f) [30]. Thus, these results suggest that the hepatoprotective effect of MSH against oxidative stress was exhibited through the activation of Nrf2 signaling pathway and the subsequent expression of its target antioxidant genes. 

### 3.5. MSH Decreases the Expression of Proinflammatory Cytokines in CCl_4_-Injured Mice and LPS-Stimulated RAW 264.7 Cells

**Figure 6:**
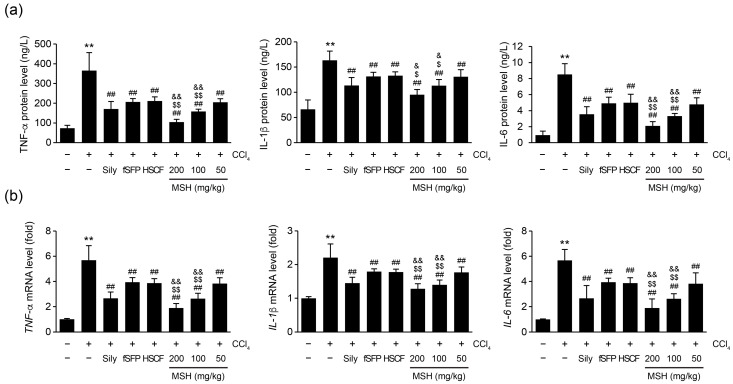
MSH inhibited the expression of proinflammatory cytokines in CCl_4_-induced liver injury mice. (**a**) Protein levels and (**b**) relative mRNA levels of tumor necrosis factor-α (TNF-α), interleukin (IL)-1β, and IL-6 in liver homogenates were quantified with ELISA and real-time PCR, respectively. Significant versus vehicle group, ** *p* < 0.01; versus CCl_4_-injected group, ^##^ *p* < 0.01; versus fSFP-treated group, ^$^ *p* < 0.05, ^$$^ *p* < 0.01; versus HSCF-treated group, ^&^ *p* < 0.05, ^&&^ *p* < 0.01.

**Figure 7:**
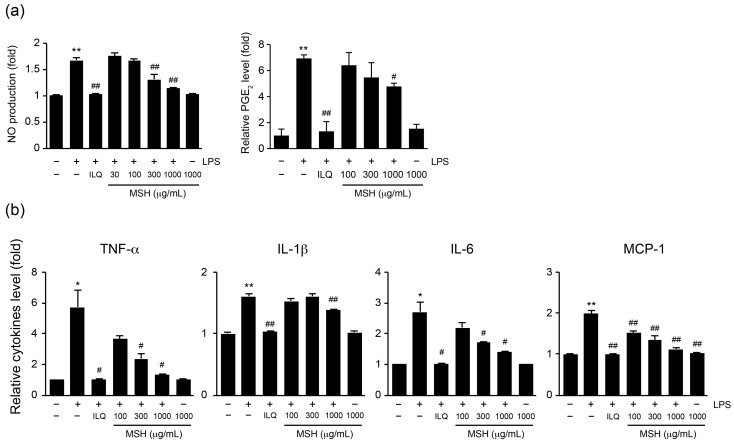
Effect of MSH on the production of inflammatory mediators in lipopolysaccharide (LPS)-stimulated RAW 264.7 cells. Cells were pretreated with 100–1000 µg/mL of MSH for 1 h, and subsequently stimulated with 1 µg/mL of LPS for 18 h. Expressions of (**a**) nitric oxide (NO), prostaglandin E_2_ (PGE_2_), (**b**) TNF-α, IL-1β, IL-6, and monocyte chemoattractant protein 1 (MCP-1) in conditioned medium were measured. All values were represented as mean ± SD of more than three separate experiments. Significant versus vehicle-control group, * *p* < 0.05, ** *p* < 0.01; versus LPS-treated group, ^#^ *p* < 0.05, ^##^ *p* < 0.01.

To investigate the effect of MSH on CCl_4_-induced hepatic inflammation, the protein and mRNA expressions of proinflammatory cytokines in hepatic tissue were measured using ELISA and real-time PCR, respectively. CCl_4_ injection significantly increased the protein and mRNA levels of proinflammatory cytokines including TNF-α, IL-1β, and IL-6 in liver homogenates. The administration of silymarin, fSFP, HSCF, and MSH (50–200 mg/kg) significantly reduced the CCl_4_-induced elevation in both mRNA and protein expressions of proinflammatory cytokines. Especially, 100 and 200 mg/kg of MSH administration showed more greater inhibitory effect than single herb extract administration (fSFP and HSCF) (Figure 6). Next, to investigate anti-inflammatory effect of MSH in macrophages, we examined anti-inflammatory effect of MSH in LPS-stimulated RAW 264.7 cells. Accordingly, pretreatment with MSH dose-dependently and significantly inhibited the expressions of inflammatory mediators including NO, PGE_2_, TNF-α, IL-1β, IL-6, and MCP-1 (Figure 7) in conditioned media.

## 4. Discussion

The liver is an essential organ with important roles in physiological processes such as energy metabolization and detoxification. However, a lack of reliable hepatoprotective agents increases the social burden of liver disease [31,32]. For this reason, numerous traditional herbal medicines and phytochemicals have been researched for the evaluation of their hepatoprotective effect. For centuries, *S. chinensis* and *H. dulcis* have been used as food and medicinal herbs in East Asia. *S. chinensis* has been used for treating symptoms such as excessive sweating, palpitation, thirst, and chronic cough, and utilized for enhancing liver function to detoxify alcohol [33]. Similarly, *H. dulcis* also has been used for treatment of liver diseases and alcohol intoxication in Chinese and Korean traditional medicine [34]. Their long history of use as both foods and medicinal herbs makes *S. chinensis* and *H. dulcis* easily obtainable ingredients for functional foods. Focusing on their ethnopharmacological evidence and accessibility, we have tried to develop a novel functional food with hepatoprotective effects by mixing the two ingredients. As part of developing a novel functional food to prevent liver disease, we evaluated the hepatoprotective effect of fSFP and HSCF (*w*:*w*; 1:1, 1:2, 1:4, 1:6, 2:1, 4:1, 6:1 and 8:1) mixtures at different combination ratios, and determined the optimal mixing ratio that exhibits the highest protective effect [24]. Based on histological observations and antioxidant capacities, we have concluded that the 1:1 ratio of MSH is the most effective among the various mixing ratios. Therefore, in present study, we aimed to evaluate the dose-dependent effect and synergistical effect of MSH, compared to single treatment of fSFP and HSCF. The results of histological analysis presented in the current study showed that MSH prevented the CCl_4_-induced degenerative changes of hepatocytes, which are marked by centrolobular necrosis, cellular swelling, and fatty vacuoles (Figure 2). Furthermore, it was observed that administration of MSH decreased the numbers of cleaved caspase 3- and cleaved PARP-positive cells in the hepatic tissue, indicating its ability to inhibit apoptosis, which is one of the forms of cell death induced by CCl_4_. Moreover, MSH significantly reduced the serum activities of ALT, AST and GGT, which released into bloodstream when hepatocytes are damaged (Figure 3). Overall, the statistically more significant preventive effects of MSH against CCl_4_-induced hepatocytes death and degeneration, compared to fSFP or HSCF single administration, provide compelling evidence for the synergistic hepatoprotective effects of MSH.

In the hepatocytes, CCl_4_-induced toxicity occurs through cytochrome P450-mediated conversion of CCl_4_ to trichloromethyl radical (CCl_3_). Further, this CCl_3_ radical is oxidized to a proxy trichloromethyl radical (CCl_3_O_2_) [35]. These forming radicals interact with lipids in liver, leading to the formation of lipid peroxidation products, such as 4-HNE and MDA. These products are well-known as potent modulator of numerous cellular processes such as cell signaling, proliferation, and death [36]. Notably, due to their high reactivity, 4-HNE and MDA can form adducts with macro biomolecules such as protein and DNA, thereby affecting their structure and function [37]. Because these mechanisms are responsible for the cytotoxicity, 4-HNE and MDA have been used for biomarkers of lipid peroxidation. Therefore, we evaluated the expression of 4-HNE and MDA in hepatic tissue to measure effect of MSH against CCl_4_-induced lipid peroxidation. Current results showed MSH successfully inhibits CCl_4_-mediated increase of 4-HNE-positive cells and MDA contents in hepatic tissue. (Figure 4). Moreover, the increased population of C11-BODIPY-positive cells in *t*BHP-stimulated HepG2 cells was diminished by MSH treatment (Figure 5). These findings suggest that MSH exhibits its protective effect on hepatocytes via defending CCl_4_-induced lipid peroxidation.

Generally, cells are equipped with antioxidant systems for protecting themselves from oxidative stress. The Nrf2 signaling pathway is a master regulator of cellular redox homeostasis. In the resting state, Nrf2 is sequestered by interacting with Kelch-like ECH-associated protein 1 (Keap1) in cytosol. However, oxidative insults degrade Keap1, and released Nrf2 is translocated to nucleus. As a transcriptional factor, Nrf2 activates transcription of antioxidant genes such as *heme oxygenase-1*, *GCLC*, *NQO1*, and *SESN2* by binding to the ARE of their promoter region [30]. In various liver disease models using high caloric diet and hepatotoxins, genetic deletion of Nrf2 increased severity and progress of liver disease [38,39,40]. On the other hand, constitutive activation of Nrf2 by genetic ablation of Keap1 confers resistance to xenobiotic-induced liver injury [41]. These findings highlight the essential role of Nrf2 in defending against liver diseases mediated by oxidative stress. Research has demonstrated that SF increases the stability and transcriptional activity of Nrf2, leading to the upregulation of antioxidant gene expression in HepG2 cells [42]. Moreover, total lignans fraction of SF exerted hepatoprotective effects against alcohol induced-liver injury via activating Nrf2/ARE signaling pathway [43]. Likewise, it has been reported HSCF exhibits a protective effect against oxidative stress-induced hepatocyte toxicity through the activation of Nrf2 [17,29]. In our investigation, MSH treatment significantly increased the ARE-driven luciferase activities and upregulated the mRNA expression of Nrf2 target genes including *NQO1*, *GCLC*, and *SESN2* in HepG2 cells. Consequently, MSH reduced *t*BHP-induced intracellular ROS productions, thereby increasing the viability of hepatocytes (Figure 5). Moreover, MSH administration also restored GSH levels and activities of SOD and CAT in CCl_4_-induced acute liver injury (Figure 4). Taken together, these results suggest that MSH protects liver by inducing restoration of endogenous antioxidant capacities via regulation of the Nrf2 signaling pathway and its target antioxidant genes expression.

Inflammation is another pathological feature propagating CCl_4_-mediated liver injury. Hepatocellular damage triggers the activation of Kupffer cells, the resident macrophages in the liver, leading to an inflammation [44]. Moreover, bacterial endotoxins such as LPS also contribute to Kupffer cell activation. The disruption of the intestinal barrier by hepatotoxic substances and their metabolites increases the bacterial translocation to liver, inducing Toll-like receptor 4-dependent Kupffer cell activation [45]. As a result, activated Kupffer cells release the cytokines and chemokines, attracting other immune cells such as neutrophils and monocytes. This immune cell infiltration further aggravates the inflammatory response in hepatic parenchyma by producing TNF-α and IL-1β [44]. TNF-α is a primary mediator of inflammation by inducing recruitment of inflammatory cells, and causes the death of hepatocytes in earliest moment of liver injury [46]. IL-1β, which has a role in modulating innate lymphoid immunity, induces the secretion of TNF-α and IL-6, and eventually contributes to liver damage [47]. In the present study, the histological observations revealed that the number of immune cells infiltrating the hepatic parenchyma was reduced by MSH administration (Figure 2). Additionally, the expressions of proinflammatory cytokine including TNF-α, IL-1β, and IL-6 in the hepatic tissue were decreased by MSH (Figure 6). To confirm our findings in animals, we further evaluated the anti-inflammatory activities of MSH in LPS-stimulated RAW 264.7 cells. As a result, MSH significantly inhibited the expressions of inflammatory mediators including NO, PGE_2_, TNF-α, IL-1β, IL-6, and MCP-1 (Figure 7). These results suggest that MSH could prevent the progression of liver disease by inhibiting the inflammatory response in hepatic tissues. Based on studies investigating the pharmacological effects of *S. chinensis* and *H. dulcis*, it can be considered that the anti-inflammatory effect of MSH is exhibited via suppression of the nuclear factor-κB (NF-κB) signaling pathway. As a transcriptional factor, NF-κB regulates the expression of genes involved in inflammation, cell survival, and proliferation, and is particularly known as a central mediator of proinflammatory cytokine induction in immune cells [48]. SF has consistently shown anti-inflammatory effects in the inflammation associated diseases including osteoarthritis, neuroinflammation, and colitis by inhibiting the NF-κB signaling pathway [49,50,51]. HSCF has also been shown to inhibit nuclear translocation of NF-κB in endotoxin-stimulated macrophage cells, thereby suppressing the production of proinflammatory cytokines [52,53].

Growing attention to the pharmacological potential of natural products has prompted active research on the chemical constituent of medicinal herbs. Among the numerous phytochemicals derived from plants, polyphenols including lignans and flavonoids are well-known for their antioxidant and anti-inflammatory properties [54,55,56]. Studies have identified lignans with a dibenzocyclooctadiene structure, such as schizandrins, gomisins, and schisantherins, are enriched in *S. chinensis* [8,57]. Among the SF-derived lignans, schizandrins have been extensively studied for their hepatoprotective effects in various experimental models of liver diseases. Notably, schizandrin A has been shown to have protective effects against high-fat and high-cholesterol diet-induced fatty liver, as well as D-gal induced liver injury [12,58]. Additionally, schizandrin B has been reported to attenuate CCl_4_-induced hepatic fibrosis by modulating Kupffer cells and hepatic stellate cells [13,14]. Furthermore, studies have reported that both schizandrin A and schizandrin B exhibit hepatoprotective effects by activating the Nrf2 signaling pathway in liver injury models induced by oxidative stress from substances such as D-gal, acetaminophen, and CCl_4_ [12,13,59]. Based on these findings, schizandrins are considered the primary chemical components of SF responsible for its hepatoprotective effects. As for HSCF, many flavonoids (e.g., myricetin, dihydromyricetin, and gallocatechin) have been identified in fruits, seeds, and peduncles of *H. dulcis* [20]. Among them, myricetin and dihydromyricetin have been extensively reported for their hepatoprotective effects [60,61]. In the case of CCl_4_-induced hepatotoxicity, it has been confirmed that myricetin effectively inhibits profibrotic response, lipid peroxidation, and inflammation in the liver of mice exposed to CCl_4_. [19,21]. Dihydromyricetin also prevented necrosis and apoptosis of hepatocytes induced by CCl_4_ injection [22]. In present study, we confirmed the presence of schizandrin and myricetin in MSH using HPLC analysis, as maker compounds of fSFP and HSCF, respectively. Considering the reported hepatoprotective effects of polyphenols derived from *S. chinensis* and *H. dulcis*, it can be speculated that the hepatoprotective effects observed in the current experiment with MSH may be attributed to the presence of these constituents.

Based on ethnopharmacological usage, the present study revealed the hepatoprotective effects of MSH against CCl_4_-induced liver injury and its synergistic action in enhancing antioxidant capacity and resolving inflammation. However, there are still several issues that need to be explored through preclinical and clinical research for the utilization as functional foods. First, identification of specific active chemical components and evaluating their pharmacological synergism in Nrf2 and NF-κB signaling pathways are needed to understand synergistic effect of MSH at the molecular level. In addition, studies confirming bioavailability of MSH and its constituents in both experimental animal and human are required for determining the efficacy and effectiveness of MSH in the body. Furthermore, a comparative analysis is needed to assess the correlation between our preclinical experimental results and their applicability to human cells. In present study, we used HepG2 cells to elucidate molecular mechanism involved in defensive effect of MSH against chemical oxidative stress inducer. While HepG2 cells have frequently been utilized to assess the potential of drug candidates, their susceptibility to chemical-induced toxicity varies from that of primary hepatocytes or stem cell-induced hepatocytes [62]. Hence, additional validation of the antioxidant effect and its mechanisms in human primary hepatocytes is necessary for potential clinical application.

## 5. Conclusions

The present investigation showed that MSH (50–200 mg/kg) alleviated the CCl_4_-induced acute liver injury by attenuating oxidative stress, inflammation, and apoptotic cell death. Especially, 100 and 200 mg/kg of MSH administration inhibited hepatic degeneration, inflammatory cell infiltration, and lipid peroxidation in hepatic tissue significantly greater than 200 mg/kg of fSFP or HSCF single administration. Moreover, 100 and 200 mg/kg of MSH administration elevated GSH level and SOD and CAT activity significantly more than 200 mg/kg of fSFP or HSCF single administration. These significantly stronger protective effects of MSH against CCl_4_-induced acute liver injury, compared to fSFP or HSCF single administration, provide compelling evidence for the synergistic hepatoprotective effects of MSH. In addition, results of in vitro assay using HepG2 and RAW 264.7 cells suggest that antioxidant and anti-inflammatory effect of MSH was exerted via activating the Nrf2 signaling pathway and inhibiting production of inflammatory mediators. Further research about efficacy in the human hepatocytes, pharmacological synergisms of chemical constituents, and bioavailability could offer promising prospects for MSH as a novel natural hepatoprotective agent against liver disease mediated by oxidative stress.

## Data Availability

Not applicable.

## Supplementary Materials

The following supporting information can be downloaded at: https://www.mdpi.com/article/10.3390/antiox12081556/s1, Figure S1: Manufacturing process of fermented Schizandrae Fructus and Hoveniae Semen cum Fructus extracts. Figure S2: High performance liquid chromatography (HPLC) analysis.
