# Supplementary material for: A Mixture of Fermented Schizandrae Fructus Pomace and Hoveniae Semen cum Fructus Extracts Synergistically Protects against Oxidative Stress-Mediated Liver Injury
Source: Antioxidants (Basel). 2023 Aug 3;12(8):1556. doi: 10.3390/antiox12081556 (PMC10451536; doi:10.3390/antiox12081556)
Supplement: Supplementary file 1 [file antioxidants-12-01556-s001.zip › antioxidants-2522800-supplementary.pdf]

antioxidants

Supplementary Information

A Mixture of Fermented Schizandrae Fructus Pomace and Hoveniae Semen cum Fructus Extracts Synergistically Protects against Oxidative Stress-Mediated Liver Injury

Jang-Soo Kim [1,†], Kyung-Hwan Jegal [2,†], Hye-Rim Park [1,3], Beom-Rak Choi [3], Jae-Kwang Kim [4,*] and Sae-Kwang Ku [1,*]

[1] Department of Anatomy and Histology, College of Korean Medicine, Daegu Haany University, Gyeongsan-si 38610, Republic of Korea; akamjnj@dhu.ac.kr (J.-S.K.); hrpark@nutracore.co.kr (H.-R.P.)
[2] Department of Korean Medical Classics, College of Korean Medicine, Daegu Haany University, Gyeongsan-si 38610, Republic of Korea; jegalkh@dhu.ac.kr
[3] Nutracore Co., Ltd., Suwon-si 16514, Republic of Korea; brchoi@nutracore.co.kr
[4] Department of Physiology, College of Korean Medicine, Daegu Haany University, Gyeongsan-si 38610, Republic of Korea
* Correspondence: kim-jk@dhu.ac.kr (J.-K.K.); gucci200@hanmail.net (S.-K.K.)
† These authors contributed equally to this work.

1. Preparation of fermented Schizandrae Fructus pomace (fSFP) and Hoveniae Semen cum Fructus (HSCF) extracts.

Raw materials (fSFP and HSCF each of 100 kg) was extracted with hot water, and then filtered. The resulting extracts were concentrated using vacuum evaporator, and dried using a spray drier on conditions indicated in manufacturing process (Figure S1).

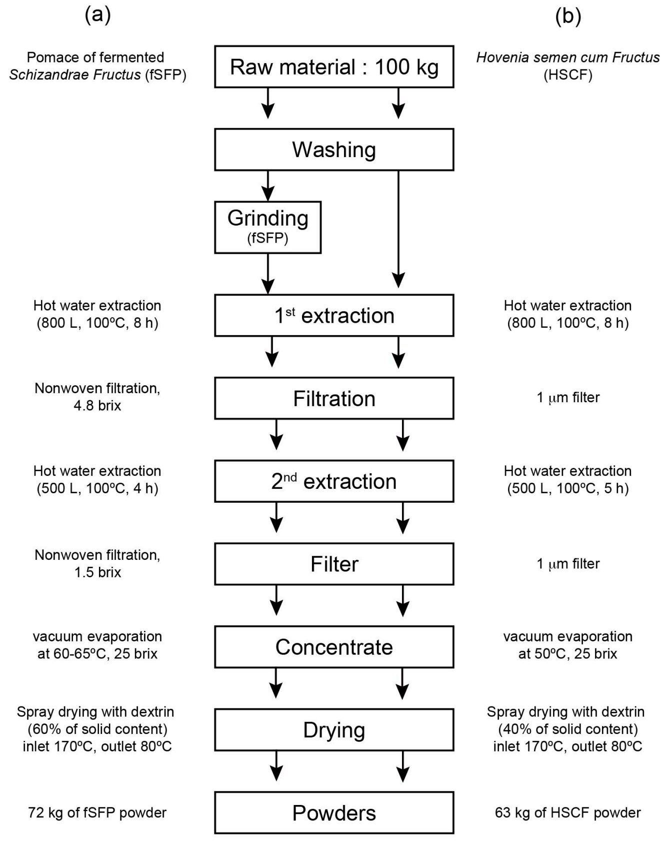

Figure S1. Manufacturing process of (a) fermented Schizandrae Fructus pomace (fSFP) and (b) Hoveniae Semen scum Fructus (HSCF).

2. Determination of schizandrin and myricetin in the mixture of fSFP and HSCF (MSH) extract

To determine schizandrin content in MSH, High-performance liquid chromatographic (HPLC) analysis was performed using Agilent HPLC system (Agilent, Waldbronn, Germany). MSH and schizandrin standard were dissolved in distilled water, and 10 μL of sample and standard were injected into the HPLC instrument, equipped with C18 column (CAPCELL PAK C_{18}, 4.6 × 250 mm, 5 μm) at 30°C column temperature. The mobile phase consisted of water with 0.05% TFA (A) and acetonitrile (B). HPLC gradient conditions were follows: 0 min (A 55%, B 45%), 40 min (A 55%, B 45%), 41–55 min (A 5%, B 95%) with the 0.7 mL/min flow rate. Absorbance at 254 nm wavelength was detected.

In addition, myricetin content in MSH was determined. MSH and myricetin standard were dissolved in 80% methanol. 20 μL of the sample and standard were injected into the HPLC instrument equipped with C18 column (Eclipse Plus C_{18}, 4.6 × 250 mm, 5 μm) at 30°C column temperature. HPLC gradient conditions were follows: 0 min (A 80%, B 20%), 25 min (A 80%, B 20%), 26 min (A 5%, B 95%), 35 min (A 5%, B 95%) with the 1.0 mL/min flow rate. Absorbance at 372 nm wavelength was detected.

(a) (b)

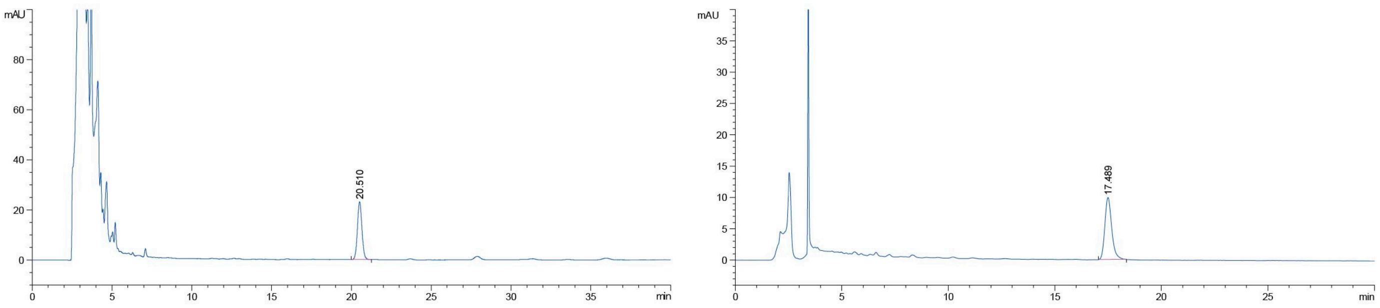

Figure S2. High performance liquid chromatography (HPLC) analysis. (a) Chromatogram of schizandrin in MSH (b) Chromatogram of myricetin in MSH.
